# Terahertz Fingerprint Metasurface Sensor Based on Temperature Variation for Trace Molecules

**DOI:** 10.3390/bios14070318

**Published:** 2024-06-24

**Authors:** Weijin Wang, Mingjun Sun, Jie Lin, Ying Xue, Yanpeng Shi

**Affiliations:** School of Integrated Circuits, Shandong University, Jinan 250100, China

**Keywords:** Terahertz, metasurface, sensing, temperature variation, plasmons

## Abstract

Terahertz (THz) spectroscopy has demonstrated significant potential for substance detection due to its low destructiveness and due to the abundance of molecular fingerprint absorption signatures that it contains. However, there is limited research on the fingerprint detection of substances at different temperatures. Here, we propose a THz metamaterial slit array sensor that exploits localized surface plasmons to enhance the electric field within the slit. The transmission peak frequency can be modulated via temperature adjustments. This method enables the detection of molecular absorption characteristics at multiple spectral frequency points, thereby achieving a specific and highly sensitive detection of characteristic analyte fingerprint spectra. Additionally, the sensor supports the detection of substances at multiple temperatures and sensitively identifies changes in their absorption properties as a function of temperature. Our research has employed temperature variation to achieve a highly sensitive and specific detection of trace analytes, offering a new solution for THz molecular detection.

## 1. Introduction

Terahertz (THz) waves possess ultra-low photon energy, which eliminates the risk of causing damaging ionization in analytes [1]. Many chemical molecules exhibit resonance and rotational frequencies within the THz range, resulting in distinct absorption resonances and intricate characteristic fingerprint spectra. Analyzing the interaction between incident THz waves and molecules provides valuable insights into molecular configuration, conformation, and environmental effects [2], thus enabling specific detection capabilities [3]. THz sensing holds significant promise for the non-destructive, rapid, and precise identification of various chemical molecules, with wide-ranging applications spanning across physics, chemistry, biomedicine, and related fields [4]. Nevertheless, a significant challenge arises from the mismatch between the THz wavelength, typically at the micrometer scale, and the absorption cross-section of molecules, which operates at the nanometer scale [5]. Most molecules exhibit weak interactions with THz waves. Traditional THz spectroscopy methods typically require large analyte quantities to observe their characteristic fingerprint spectra, leading to low sensing sensitivity [6,7]. Enhancing the sensing sensitivity of analytes in practical applications has become imperative.

The application and sensing of high-energy explosive has been one of the international research hotspots. Hexogen (RDX), also known as cyclonite, is a powerful military explosive. It is a white crystalline powder and is widely used in various fields such as military, aerospace, mining, and chemical industries due to its high explosive speed and power [8]. It is almost impossible to detect through X-ray imaging, necessitating the development of new non-contact and non-destructive techniques for effective detection. THz waves, due to their non-destructive nature, offer unique advantages for RDX detection. The broad frequency range and weak absorption of RDX fingerprints limit the practical applications of THz sensing. Enhancing the sensitivity of THz sensing for RDX is the primary focus of current research. HNIW (CL-20), chemically known as 2,4,6,8,10,12-hexanitro-2,4,6,8,10,12-hexaazaisowurtzitane, is a highly energetic and potent non-nuclear elementary explosive. It has wide-ranging applications in military and civilian sectors, garnering significant attention from researchers. CL-20 undergoes an irreversible ε–γ phase transition induced by temperature, a process that is closely linked to its explosive behavior. The precise detection of CL-20 and its phase transition are crucial to ensure its safe utilization [9,10]. Unfortunately, achieving a rapid and highly sensitive sensing of CL-20 and its phase transition process remains challenging. Specifically, detecting fingerprints at different temperatures before and after the ε–γ phase transition of CL-20 presents significant difficulties.

In previous studies, researchers have proposed various artificially designed metasurface structures, including open-ring resonators [11], metal gratings [12], photonic crystal structures [13], waveguide configurations [14], and structures based on bound states in the continuum [15,16], to achieve heightened sensitivity in material detection [17,18,19]. The majority of these structures are limited to detecting material absorption characteristics at individual frequency points via resonance peaks. They cannot accurately analyze the absorption spectra curve within a specific frequency range to identify particular substances [20]. In recent years, researchers have employed strategies such as structural scanning, angle scanning, stretchable flexible materials, and adjustable graphene to modulate the resonance peak frequency [21]. This advancement allows for the detection of characteristic absorption curves within a defined frequency band, facilitating material analysis and specific substance identification [22,23]. Nonetheless, limitations persist, including narrow sensing frequency bands and the inconvenience of frequency modulation strategies. Current research on the spectral response and sensing capabilities of metasurfaces at varying temperatures remains inadequate.

In this paper, we propose a THz metamaterial nano-slit array sensor based on temperature variation to enhance the detection of molecular fingerprint spectra. The sensor utilizes the temperature-sensitive semiconductor material InSb to form the nano-slit array structure, successfully generating localized surface plasmons (LSPs) [24]. When a surface plasmon is confined to a particle of a size comparable with the wavelength of light, the particle’s free electrons participate in the collective oscillation, and it is termed an LSP. LSPs significantly enhance the electric field near the particle’s surface, with the maximum enhancement at the surface rapidly diminishing with distance. The particle’s optical extinction peaks at the plasmon resonance frequency. This extinction peak depends on the refractive index of the surrounding medium [25]. The sensor achieves localized electric field enhancement based on LSP, significantly improving sensing sensitivity [26,27]. InSb is a frequently utilized temperature-sensitive material. As the temperature increases, both the real and imaginary components of the InSb dielectric constant in the terahertz band significantly and continuously increase. This leads to alterations in the metasurface resonance frequency [28]. When the temperature varies from 275 K to 520 K, the resonance peak frequency gradually shifts from 0.78 THz to 1.6 THz, with a change in amplitude of 0.82 THz. By continuously changing temperatures, the resonance peak can be located at different frequency points. This allows for the sensitive sensing of molecular absorption features at multiple frequencies and the formation of an envelope curve depicting characteristic absorption spectra. Taking RDX detection as an example, this sensor has a detection limit of 1.61 μg/cm^2^. The proposed sensor can specifically identify trace substances. It provides a reliable approach for trace molecule sensing using THz technology [29]. In addition, the sensor can operate at multiple temperatures. By altering the slit length to modify the resonance frequency at a constant temperature, characteristic absorption spectra at multiple temperatures can be detected. By coating the sensor surface with CL-20, distinct characteristic absorption curves of CL-20 were detected at 298 K and 453 K. This enables the observation of changes in CL-20 absorption features before and after the ε–γ phase transition. The sensor can detect changes in molecular fingerprint spectra with temperature. This advances the application of THz sensing in monitoring temperature-dependent absorption characteristics of substances.

## 2. Structure and Design

**Figure 1:**
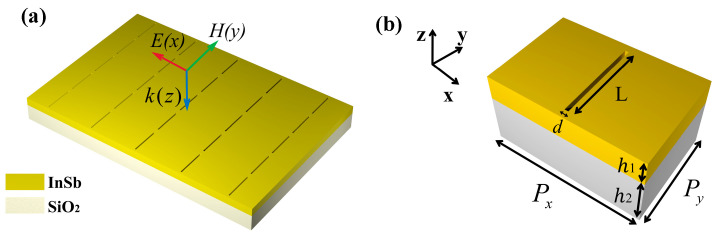
(**a**) Structure diagram of the metasurface sensor. (**b**) Metasurface cell and its structural parameters.

Figure 1a illustrates the structural diagram of the THz metamaterial nano-slit array sensor. THz waves, polarized along the x-axis, propagate vertically into the metamaterial. The substrate material utilized is silicon dioxide. The relative permittivity of SiO₂ shows no significant differences at various operating temperatures. We approximate that the relative permittivity of SiO₂ remains constant at 1.73 across all operating temperatures [30,31,32]. The nano-slit array structure comprises the temperature-sensitive InSb, whose dielectric constant can be approximated by the Drude model as [33].

(1) $$\varepsilon (\omega )=\varepsilon_{\infty }-\frac{\omega_{p}^{2}}{\omega^{2}+i\gamma \omega }$$

In Equation (1), $\varepsilon_{\infty }$ = 15.68 denotes the dielectric constant at infinite angular frequency, ω represents the angular frequency, $\omega_{p}=\sqrt{Ne^{2}/\varepsilon_{0}m^{*}}$ denotes the plasma frequency, γ = 0.1π THz signifies the damped vibration frequency, N denotes the intrinsic carrier concentration, $\varepsilon_{0}$ denotes the vacuum dielectric constant, e represents the electron charge, and $m^{*}=0.015m_{e}$ ($m_{e}$ denotes the free electron mass) represents the effective mass of the free carriers [34].

N can be expressed by the following relation [35]:

(2) $$N=5.76\times 10^{20}T^{1.5}exp\left(-0.26/2k_{B}T\right)$$

In Equation (2), $k_{B}$ denotes the Boltzmann constant, and T represents the temperature in Kelvin. Hence, the intrinsic carrier concentration N of InSb is influenced by the external temperature, subsequently affecting its dielectric constant. Therefore, InSb serves as a temperature-sensitive semiconductor material, facilitating the convenient modulation of the sensor’s resonance peak via changes in the external environment, ultimately leading to the formation of a broadband transmission envelope.

Figure 1b depicts the unit cell of the metamaterial. The geometric parameters of the unit cell are set as follows: period $P_{x}$ = 120 μm and $P_{y}$ = 80 μm; slit width d = 3 μm and length L = 68 μm; thickness of InSb and slit $h_{1}$= 20 μm; and thickness of the SiO_2_ substrate $h_{2}$ = 40 μm. To investigate the performance of the sensor, the optical characteristics of the metamaterial are simulated using three-dimensional finite-difference time-domain (FDTD) software. In the simulation, perfect matching layers are applied along the z-direction, while periodic boundary conditions are applied along the x- and y-directions.

## 3. Result and Discussions

**Figure 2:**
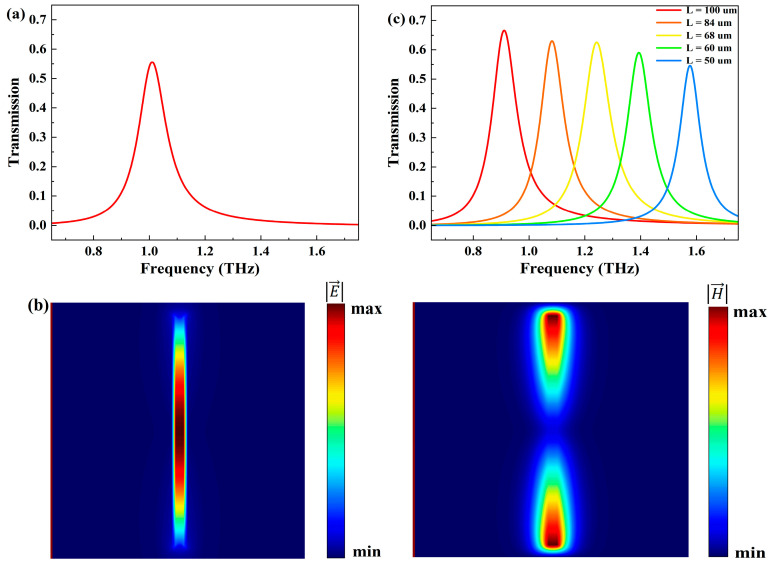
(**a**) Transmission spectrum of the metasurface at T = 310 K. (**b**) Electric and magnetic field distributions at f = 1 THz. (**c**) Transmission spectra for different slit lengths L at T = 360 K.

Figure 2a depicts the transmission spectrum of the proposed metasurface sensor when THz waves, polarized along the x-axis, vertically impinge upon the metasurface at an ambient temperature of 310 K. As observed from Figure 2a, a prominent resonance transmission peak is evident at 1 THz. Meanwhile, Figure 2b illustrates the distribution of electric and magnetic fields at the resonance frequency. The electric field (x-z plane) is entirely localized at the slit and is significantly enhanced. The magnetic field is localized at the ends of the slit [36]. Such a field distribution markedly enhances the interaction between THz waves and analytes, thereby enhancing the sensitivity of the sensor [37].

To comprehensively understand the impact of slit length L on the resonance transmission peak, the transmission spectra of sensors with different L ranging from 50 μm to 100 μm were analyzed. Simultaneously, while adjusting the slit length L, the distance between slits (the period $P_{y}$) was adjusted to prevent adjacent slits from contacting each other. Specifically, when L = 50, 60, 68, 84, 100 μm, the corresponding periods $P_{y}$ were 70, 80, 80, 110, and 120 μm, respectively. As depicted in Figure 2c, with increasing L, the resonance peak frequency gradually decreases, while the transmission peak value slightly increases [38]. The slit length L can influence the resonance frequency and alter the position of the resonance peak. The change in transmission peak value can be explained by the dielectric properties of InSb. As the frequency decreases, the extinction coefficient k of InSb gradually increases. This significantly suppresses THz transmission, thereby reducing the peak transmission.

**Figure 3:**
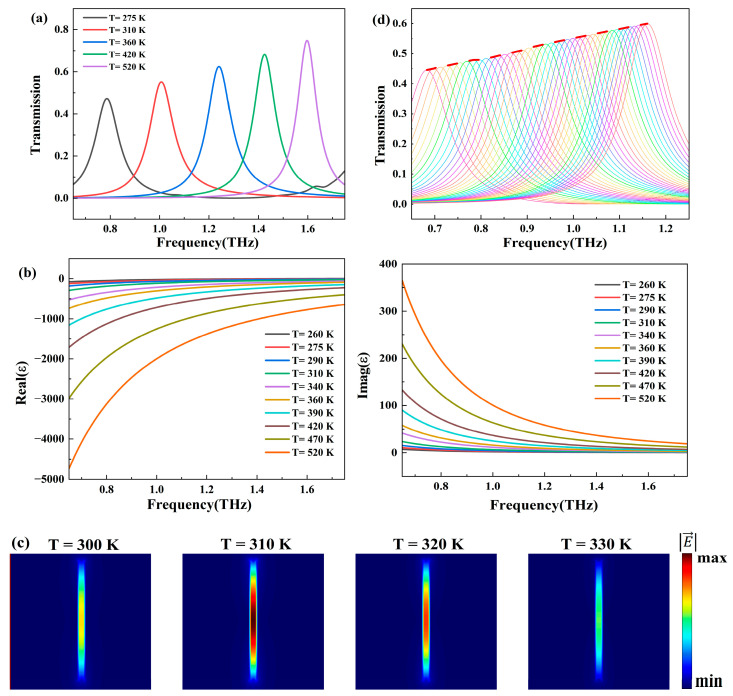
(**a**) Transmission spectra of the metasurface within the ambient temperature range of 275 K to 520 K. (**b**) Dielectric constant of InSb at different ambient temperatures. (**c**) The electric field distribution of the metasurface at 1 THz from 300 K to 330 K. (**d**) Transmission spectra of the metasurface and its envelope curves (the red curve) within the ambient temperature range of 260 K to 340 K (increment of 2 K).

To characterize the spectral response of the sensor, the relationship between the structural transmission curve and temperature T was investigated. Figure 3a illustrates the variation of the sensor’s transmission spectra with temperature T ranging from 275 K to 520 K. As the temperature increases, the resonance angle experiences a blueshift, and the transmission peak value continuously increases. Under slit length L = 68 μm, the resonance peak can shift from 0.78 THz to 1.6 THz, achieving an ultra-wideband shift of 0.82 THz. Figure 3b demonstrates the relationship between the dielectric constant of InSb and temperature, explaining the variation in resonance frequency and transmission peak value [39]. Figure 3c shows the electric field distribution of the metasurface at 1 THz from 300 K to 330 K. The electric field within the slits is significantly enhanced, with the maximum electric field intensity occurring at 310 K. Both increasing and decreasing the temperature lead to a reduction in electric field intensity, corresponding to changes in transmittance at 1 THz for the metasurface. We selected a room temperature range of 260 K to 340 K for molecular fingerprint sensing [40]. Figure 3d presents the transmission spectra (from 260 K to 340 K, with a step size of 2 K) and corresponding metasurface envelope curves. The resonance angles of the transmission spectra at different temperatures were extracted and further fitted into the envelope curves through interpolation (as shown by the red curve in the figure) [41]. With a temperature variation of only 80 K, this envelope curve can cover a broad frequency range from 0.68 THz to 1.16 THz, which is advantageous for the characteristic fingerprint sensing of substances. In this scenario, when the analyte is placed at the slit of the metasurface, an absorption peak matching the fingerprint of the analyte will be observed in the envelope curve [42,43].

**Figure 4:**
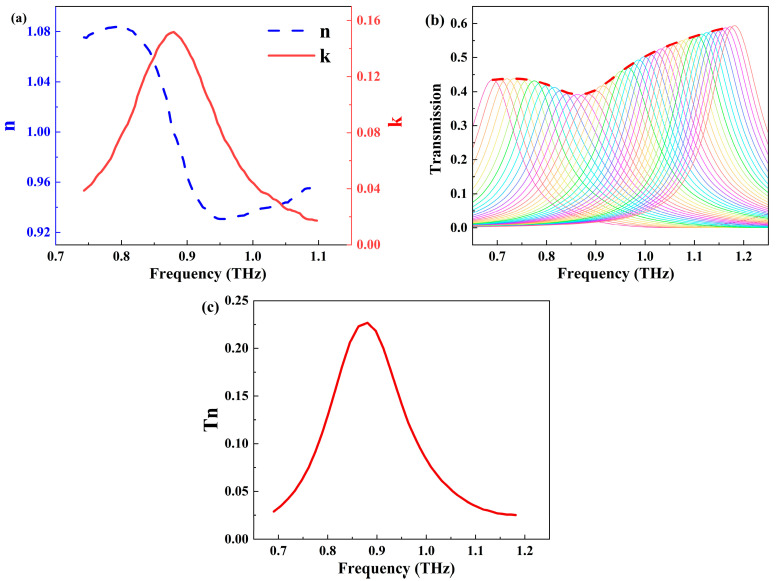
(**a**) Refractive index n and extinction coefficient k of RDX in the THz range. (**b**) Transmission spectra of the metasurface covered with 8.03 μg/cm^2^ RDX as a function of ambient temperature (260–340 K) and the corresponding envelope curves (the red curve). (**c**) Envelope curves of the transmission spectra of the metasurface after normalization.

To study its sensing performance, we covered the sensor surface with RDX. Based on the existing THz time-domain spectroscopy analysis results of RDX, we obtained the refractive index n and extinction coefficient k of RDX in the THz range [44], as shown in Figure 4a. Due to weak intermolecular interactions and lattice vibrations, RDX exhibits a weak absorption peak at 0.88 THz [45]. Additionally, the absorption peak of RDX is relatively broad, necessitating a sufficiently wide sensing frequency bandwidth. Figure 4b illustrates the transmission spectra of the metasurface covered with 8.03 μg/cm^2^ RDX as a function of temperature (260–340 K) and the corresponding envelope curves. Because RDX exhibits excellent chemical stability at room temperature and can stably exist for long periods below 400 K, detection at temperatures ranging from 260 K to 340 K is reasonable. With increasing temperature, a significant blueshift in the resonance angle is observed, and a distinct absorption peak appears at 0.88 THz in all spectra. The maximum transmission rate at the same frequency point for different temperatures was extracted, and the results were further fitted into envelope curves through interpolation. It can be observed that the envelope curve exhibits the minimum transmission rate at 0.88 THz and forms an envelope peak at this frequency, corresponding to the fingerprint spectrum of RDX [46]. Let the envelope curve of the sensor without RDX coverage be denoted as T0 and the envelope curve of the sensor covered with 8.03 μg/cm^2^ RDX be denoted as Ts. The change in transmission rate was normalized to Tn = (T0 − Ts)/T0. The normalized envelope curve Tn shown in Figure 4c closely follows the trend of the extinction coefficient k of RDX. This indicates that our proposed nano-slit array sensor can accurately amplify and detect the characteristic absorption fingerprint of RDX [47,48], achieving a specific and highly sensitive sensing of RDX.

**Figure 5:**
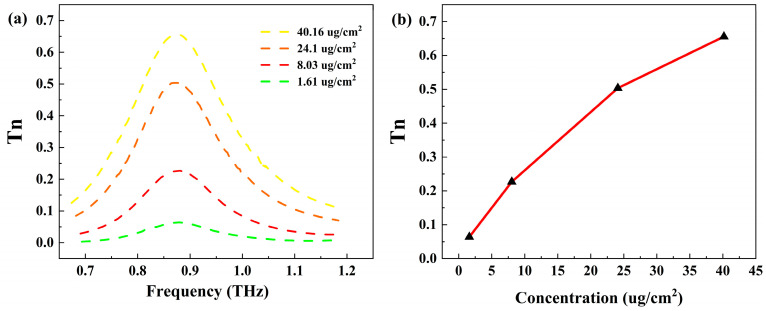
(**a**) Normalized envelope curves deposited with different concentrations of RDX on the metasurface. (**b**) Relationship between the normalized transmission rate change at 0.88 THz and the RDX concentration.

To further investigate the system’s capability to detect trace analytes, we covered the metasurface with different concentrations of RDX. Figure 5a illustrates the normalized envelope curves deposited with varying concentrations of RDX on the metasurface. As the RDX concentration increases from 1.61 μg/cm^2^ to 40.16 μg/cm^2^, the corresponding normalized transmission rate change also gradually increases. Figure 5b demonstrates the relationship between the normalized transmission rate change at 0.88 THz and the RDX concentration in the range of 1.61 μg/cm^2^ to 40.16 μg/cm^2^. The linear fitting equation of the curve in the figure is approximately y = 0.01519x + 0.08182, where y represents the normalized transmission rate change at 0.88 THz and x denotes the RDX concentration in μg/cm^2^. The correlation coefficient of this fitting curve is 0.98, indicating a good linear correlation between the normalized transmission rate change at 0.88 THz and the RDX concentration. In our experiments, the minimum RDX concentration featured a clearly observable envelope absorption peak, and the detection limit of our sensor was 1.61 μg/cm^2^. These studies suggest that RDX content can be detected based on the normalized transmission rate change of the envelope peak, enabling a quantitative sensing of RDX with a very low detection limit.

**Figure 6:**
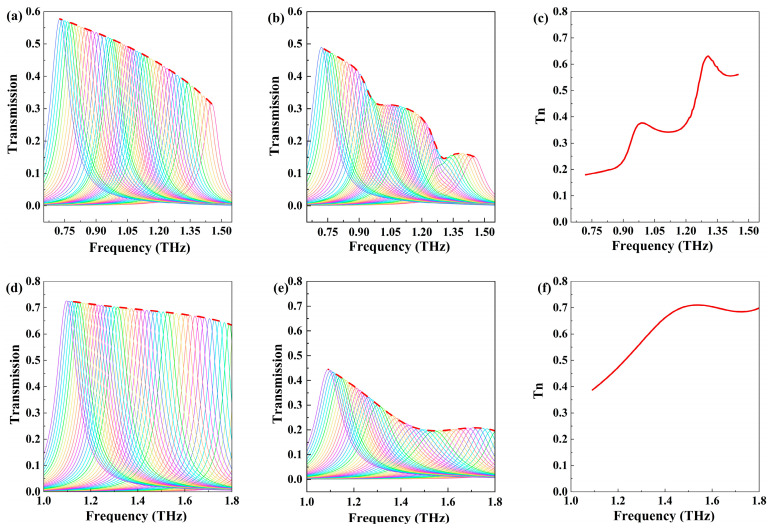
(**a**) Metasurface transmittance spectra and envelope curve of slit length L from 36 μm to 100 μm at 298 K. (**b**) Metasurface transmittance spectra and envelope curves after covering trace CL-20 at 298 K. (**c**) The envelope curve after normalization at 298 K. (**d**) Metasurface transmittance spectra and envelope curve at 453 K. (**e**) Metasurface transmittance spectra and envelope curves after covering trace CL-20 at 453 K. (**f**) The envelope curve after normalization at 453 K.

In addition, the sensor can track the characteristic absorption spectra changes of CL-20 before and after the temperature-induced phase transition. Previous studies have demonstrated that CL-20 can undergo an irreversible ε–γ phase transition triggered by temperature variation. At 298 K, the ε-CL-20 phase exhibits two absorption peaks at 1.31 THz and 0.99 THz. As the temperature increases to 453 K, CL-20 transitions into the γ phase. The characteristic absorption peaks at 1.31 THz and 0.99 THz diminish, while a strong absorption occurs at 1.53 THz [9,10]. When the ambient temperature of the metasurface is maintained at 298 K, altering the slit length L results in a redshift in resonance frequency and a slight increase in transmission peak value. Different slit lengths are employed across various regions of the metasurface to encompass the resonance inclination angles of different frequency positions, forming corresponding envelope curves [22]. Figure 6a illustrates the transmission spectra and their envelope curve (depicted in red) for slit lengths ranging from 100 μm to 36 μm. This envelope curve spans the 0.72–1.45 THz range. As the slit length L varies, the period $P_{y}$ changes with it, ensuring that the distance between the edges of two adjacent slits in the y-direction remains at 40 μm. Figure 6b displays the transmission spectra and their envelope curve (depicted in red) following the introduction of trace CL-20. Notably, distinct absorption peaks emerge at 1.31 THz and 0.99 THz, aligning with the position of the characteristic absorption peak of ε-CL-20. Figure 6c presents the normalized envelope curve, effectively describing the absorption characteristics of ε-CL-20 at 298 K. As the ambient temperature rises to 453 K, Figure 6d showcases the transmission spectra and their envelope curve, with only the transmission spectra from 1 THz to 1.8 THz being displayed, omitting the formant at higher frequency positions. Figure 6e exhibits the transmission spectra and its envelope curve following the introduction of an equal content of CL-20, while Figure 6f represents the normalized envelope curve. At 453 K, the envelope curve exhibits strong absorption at 1.53 THz, while the absorption peak at 1.31 THz disappears. This is consistent with the absorption characteristics of γ-CL-20, effectively describing the absorption features of CL-20 at 453 K. The comparative analysis of the transmission spectra and envelope curves at 298 K and 453 K, covering trace amounts of CL-20, shows a shift in the characteristic absorption peak frequencies from 0.99 THz and 1.31 THz to 1.53 THz. This allows for a clear observation of CL-20’s absorption characteristics before and after the phase transition, facilitating the sensing and study of its phase transition mechanism. These findings validate the system’s capability to perform highly sensitive sensor detection of material feature absorption fingerprints at multiple temperatures and track changes in material feature absorption fingerprints with temperature.

## 4. Conclusions

In summary, we propose a nano-slit array sensor based on temperature variation that is capable of a highly sensitive and specific sensing of molecular fingerprints. By integrating structural parameter scanning, this sensor allows for the detection of substance characteristics at various temperatures. The metasurface consists of periodically arranged InSb slits, with SiO_2_ as the substrate. When THz waves are vertically incident on the metasurface, LSPs are successfully excited, leading to the localized enhancement of the electric field at the slits. This enhanced electric field significantly amplifies the interaction between the analyte and THz waves, effectively improving sensing sensitivity. With the variation in temperature, the dielectric properties of the temperature-sensitive InSb continuously vary, causing the transmission resonance angle to shift, thereby generating transmission envelope curves covering a wide frequency range. Based on the temperature-scanning strategy, we successfully delineate the characteristic fingerprint spectra of RDX, enabling qualitative and quantitative sensing of RDX with a detection limit of 1.61 μg/cm^2^. Simultaneously, employing the structural parameter scanning method and adjusting the slit length L, we successfully depicted the characteristic fingerprint spectra of ε-CL-20 at 298 K and γ-CL-20 at 453 K. This approach achieved a high sensitivity detection of CL-20 absorption features at different temperatures, allowing for the observation of the variation in CL-20 ε–γ phase transition absorption characteristics. Our research indicates that the proposed temperature-scanning metasurface sensor holds significant potential for the wideband fingerprint sensing of trace analytes, thus promoting the development of THz sensing in various fields, including civilian and military security, biomedicine, and beyond.

## Data Availability

The data underlying the results presented in this paper may be obtained from the authors upon reasonable request.
